# Case report: Spontaneous rupture of leiomyosarcoma uteri 8 months after primary laparoscopic surgery of STUMP

**DOI:** 10.3389/fmed.2024.1407546

**Published:** 2024-05-31

**Authors:** Marija Bicanin-Ilic, Igor Ilic, Aleksandra Dimitrijevic, Srdjan Mujkovic, Nikola Jovic, Dejana Rakic, Neda Arsenijevic, Tamara Nikolic-Turnic, Goran Balovic, Andjela Peric, Aleksandra Mitrovic, Aleksandar Nikolov

**Affiliations:** ^1^Department of Gynecology and Obstetrics, Faculty of Medical Sciences, University of Kragujevac, Kragujevac, Serbia; ^2^Clinic of Gynecology and Obstetrics, University Clinical Center Kragujevac, Kragujevac, Serbia; ^3^Department of Radiology, University Clinical Center Kragujevac, Kragujevac, Serbia; ^4^Department of Pharmacy, Faculty of Medical Sciences, University of Kragujevac, Kragujevac, Serbia; ^5^N.A. Semashko Public Health and Healthcare Department, F.F. Erismann Institute of Public Health, I.M. Sechenov First Moscow State Medical University (Sechenov University), Moscow, Russia; ^6^Department of Surgery, Faculty of Medical Sciences, University of Kragujevac, Kragujevac, Serbia; ^7^Center of Pediatric Surgery, University Clinical Center Kragujevac, Kragujevac, Serbia

**Keywords:** STUMP, leiomyosarcoma, leiomyosarcoma rupture, STUMP progression, STUMP recurrence, laparoscopy, uterus

## Abstract

**Introduction:**

Leiomyosarcoma (LMS), together with smooth muscle tumors of uncertain malignant potential (STUMP) and benign leiomyomas, belongs to a heterogeneous group of uterine neoplasms. According to the World Health Organization, tumors originating from uterine smooth muscle fibers are the second most frequent tumors. It is challenging to distinguish between STUMP and LMS because of an overlap of symptoms, lack of a precise definition, and unequivocal information obtained using imaging diagnostic methods. Following myomectomy or hysterectomy with laparoscopic or laparotomy surgery and a definitive histological diagnosis of STUMP, the course of treatment is determined by the need to preserve fertility. In 2014, the U.S. Food and Drug Administration published an alert that unprotected laparoscopic morcellation is correlated with a 3-fold higher likelihood of dissemination of malignant cells and disease progression. Unprotected morcellation was independently associated with a higher risk of disease recurrence after demolition or conservative surgery, with a relative risk of 2.94.

**Conclusion:**

Hematoperitoneum resulting from the spontaneous rupture of a uterine tumor is a rare gynecological emergency, with very few cases reported in the last decade.

## 1 Introduction

Leiomyosarcoma (LMS), together with smooth muscle tumors of uncertain malignant potential (STUMP) and benign leiomyoma, belongs to a group of heterogeneous mesenchymal uterine neoplasms (1). This group of tumors originating from uterine smooth muscle fibers is the second most frequent tumor category, affecting 70% of women in their lifetime and causing significant quality of life and economic problems (2, 3). The common symptoms caused by this group of tumors include dysmenorrhea, abnormal uterine bleeding, back pain, pelvic pressure, urinary urgency, anemia, and infertility (1, 3, 4). These tumors are classified into three groups according to mitotic activity, proliferative capacity, cytological atypia, and tumor necrosis or coagulation. The Stanford criteria, proposed by Bell et al., are an attempt to objectivize the pathohistological diagnosis of malignant smooth muscle tumors. By these criteria, LMS must satisfy at least two of the three diagnostic indicators: diffuse moderate-to-severe atypia, a mitotic count of at least 10 mitotic figures (MFs)/10 high power fields (HPFs), and tumor cell necrosis (5). In contrast, STUMP usually only exhibits one indicator (2, 6, 7). Diagnosis should be obtained by a dedicated pathologist with a high level of experience in gynecological oncology. It is challenging to distinguish between STUMP and LMS because of an overlap of symptoms, lack of a precise definition, complexity of pathophysiological findings, and unequivocal information obtained using imaging diagnostic methods. According to the World Health Organization definition, any uterine smooth muscle tumor with features indicating a potential malignancy that does not fulfill the criteria for LMS or leiomyoma may be diagnosed as a STUMP (8). LMSs are rare malignant tumors, representing 1–3% of all malignant uterine tumors, with an unfavorable prognosis, early metastases, and high rates of recurrence (9, 10). Total en bloc hysterectomy is the treatment of choice for LMS (without morcellation), while adjuvant radiotherapy is not recommended. There is no consensus on the use of adjuvant chemotherapy (10). The clinical symptoms and morphological characteristics of malignant and benign uterine tumors widely overlap, making preoperative diagnosis difficult (11). There is also a significant overlap in terminologies, such as STUMP, atypical leiomyoma, atypical leiomyoma with a low risk of recurrence, and atypical leiomyoma with low malignant potential, which contributes to confusion and unequivocal diagnosis (11). Usually, patients with symptom onset require surgical intervention that depends on the patient’s age, fertility requirements, tumor type, and surgical skill. Although imaging studies can provide useful information, they are not sufficient to distinguish between the malignant and benign nature of the tumor. Therefore, histopathological STUMP or LMS diagnosis is most often determined by a pathologist postoperatively (3, 8, 9). Diagnoses are usually made incidentally after patients have undergone fibroid surgery (12). There are no specific postoperative management protocols for STUMP, as they are extremely rare tumors. Data regarding recurrence and metastasis are obtained from case studies, and there is a lack of data regarding their biological behavior and long-term outcomes. Additionally, there is heterogeneity in histopathological and imaging features (4, 13).

After patients undergo laparoscopy or laparotomy (myomectomy or hysterectomy) and a definitive histopathological STUMP diagnosis is made, further medical treatment depends on the requirements for fertility preservation (4). Myomectomy is an acceptable choice for patients who want to preserve fertility, but surgical radicalization should be proposed when childbearing is completed (3, 11). The reported recurrence rate after primary surgical resection varies from 7 to 36.4%, and recurrence can present as STUMP or LMS (14). A literature review reported that 5 of 76 (6.6%) patients who underwent myomectomy experienced a relapse, with a follow-up interval ranging from 0.1 to 18 years. Regular follow-ups and monitoring are required if patients decide to undergo conservative treatments. Ultrasonography of the minor pelvis and abdomen, chest radiography, and a gynecologic examination should be performed to rule out new masses and signs of hemorrhage (14). Annual whole abdominal computed tomography should be performed together with magnetic resonance imaging (MRI), especially in patients who undergo unprotected laparoscopic morcellation. In 2014, the U.S. Food and Drug Administration published an alert that this specific procedure is correlated with a 3-fold higher likelihood of dissemination of malignant cells and disease progression (4, 14). Unprotected morcellation was independently associated with a higher risk of disease recurrence after demolition or conservative surgery, with a relative risk of 2.94 (4).

Hematoperitoneum resulting from the spontaneous rupture of a uterine tumor is a rare gynecological emergency, with very few cases reported in the last decade (1). Gynecological hematoperitoneum is usually caused by an ectopic pregnancy, ruptured ovarian cysts, torsion, or trauma (15, 16).

## 2 Case report

A 49-year-old Caucasian woman was admitted to the Clinical Center Kragujevac, Department of Gynecology and Obstetrics. She was referred by a private practice gynecologist and presented with symptoms of prolonged metrorrhagia and severe anemia. The patient denied taking any medications, food allergies, or previous surgical interventions. She was diagnosed with multiple sclerosis. She had been prescribed corticosteroid therapy but avoided taking medications and follow-up visits; she was symptom-free at the time. Family history: Her mother had a malignant ovarian tumor. Personal history: Our patient had menarche at the age of 13 years, with regular periods every 30 days lasting 7 days. The last period started 1 month prior and was still ongoing. The patient had two vaginal deliveries and no miscarriages.

Clinical findings on the first day of hospitalization: The patient had abnormal uterine bleeding.

Abdominal probe ultrasound findings: The entire minor pelvis was filled with an isoechogenic non-uniform myometrial lesion 133 × 119 × 120 mm in diameter that involved more than 50% of the myometrium, with moderate acoustic shadowing and a circumferential vascular pattern of the lesion.

Laboratory findings on admission day: leukocytes, 8.8 × 10^9^/L; erythrocytes, 2.12 × 10^9^/L; hemoglobin, 50 g/L; hematocrit, 0.162 L/L; mean corpuscular volume, 76.6 fL; mean corpuscular hemoglobin, 23.7 pg.; mean corpuscular hemoglobin concentration, 309 g/L; red cell distribution width, 18.6%; and platelets, 716 × 10^9^/L.

The patient was treated at the hospital with three doses of red blood cells and resuspended erythrocytes for moderate anemia.

Laboratory findings after therapy/hematology: leukocytes, 8.61 × 10^9^/L; erythrocytes, 3.36 × 10^12^/L; hemoglobin, 89 g/L; and hematocrit, 0.297 L/L; mean corpuscular volume, 88.4 fL.

Considering the patient’s age (49 years) and size and ultrasound image characteristics of the tumor, together with no desire for fertility, our surgeon proposed open hysterectomy as surgical treatment.

The patient agreed to the proposed abdominal hysterectomy; however, she reconsidered her decision and sought a second opinion. The patient was discharged from the hospital for personal reasons. Our patient underwent laparoscopic myomectomy (unprotected) in a private surgical clinic performed by an expert gynecological surgeon. Histopathological findings showed that the tumor removed laparoscopically was a STUMP. The patient was advised by her surgeon to undergo another surgery—a hysterectomy; however, she did not accept the treatment proposal.

During the first and second postoperative follow-up visits, ultrasonography revealed an isoechoic lesion at the postoperative scarring site on the uterus, which was described by the radiologist as a hematoma. The patient received conservative treatment with antibiotics and anti-anemia therapy.

Eight months after the laparoscopic surgery, the patient was admitted to the hospital by a gynecologist because of severe abdominal pain, nausea, and vomiting.

Gynecological examination revealed that the entire minor pelvis and lower and middle abdomen were filled with a painful solid tumor mass with limited mobility.

Abdominal ultrasonography revealed a large hyperechogenic tumor with an unclear anterior surface and free fluid in the abdominal cavity.

**Figure 1:**
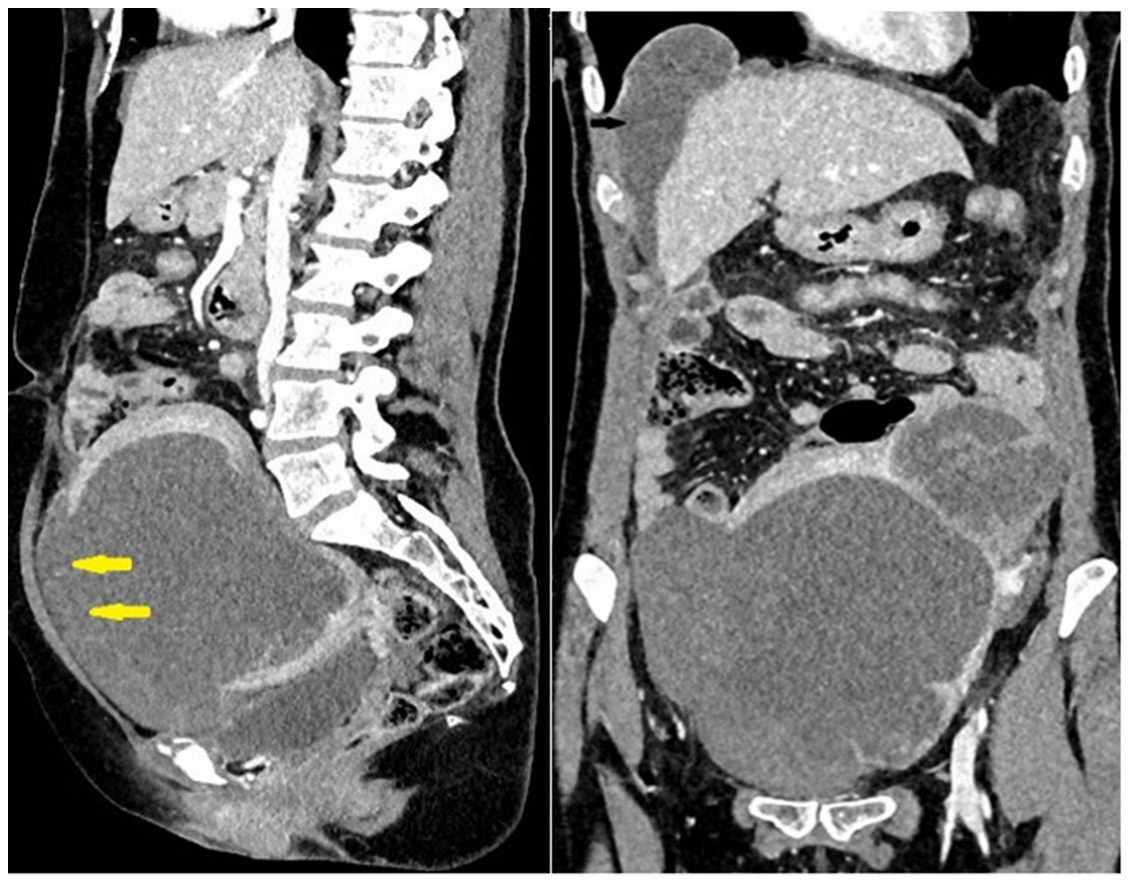
Large uterine tumor rupture (yellow arrows) with hemoperitoneum. Metastases in the lung and liver.

Urgent multidetector computed tomography (MDCT) of the abdomen and pelvis showed an enlarged uterus with a tumor mass of 110 × 100 mm in diameter, a lobulated contour, and disruption of the anterior wall of the uterine contour (consistent with uterine rupture), which caused hematoperitoneum. A cystic lesion in the liver with small solid parts and a septum 38 mm in diameter were also found. A few cyst-like focal lesions up to 20 mm in size were found in the lungs (Figure 1).

The patient was informed about the urgency of the medical condition and the need for surgical intervention. After the patient provided signed informed consent and adequate preoperative preparation, an open laparotomy was performed.

**Figure 2:**
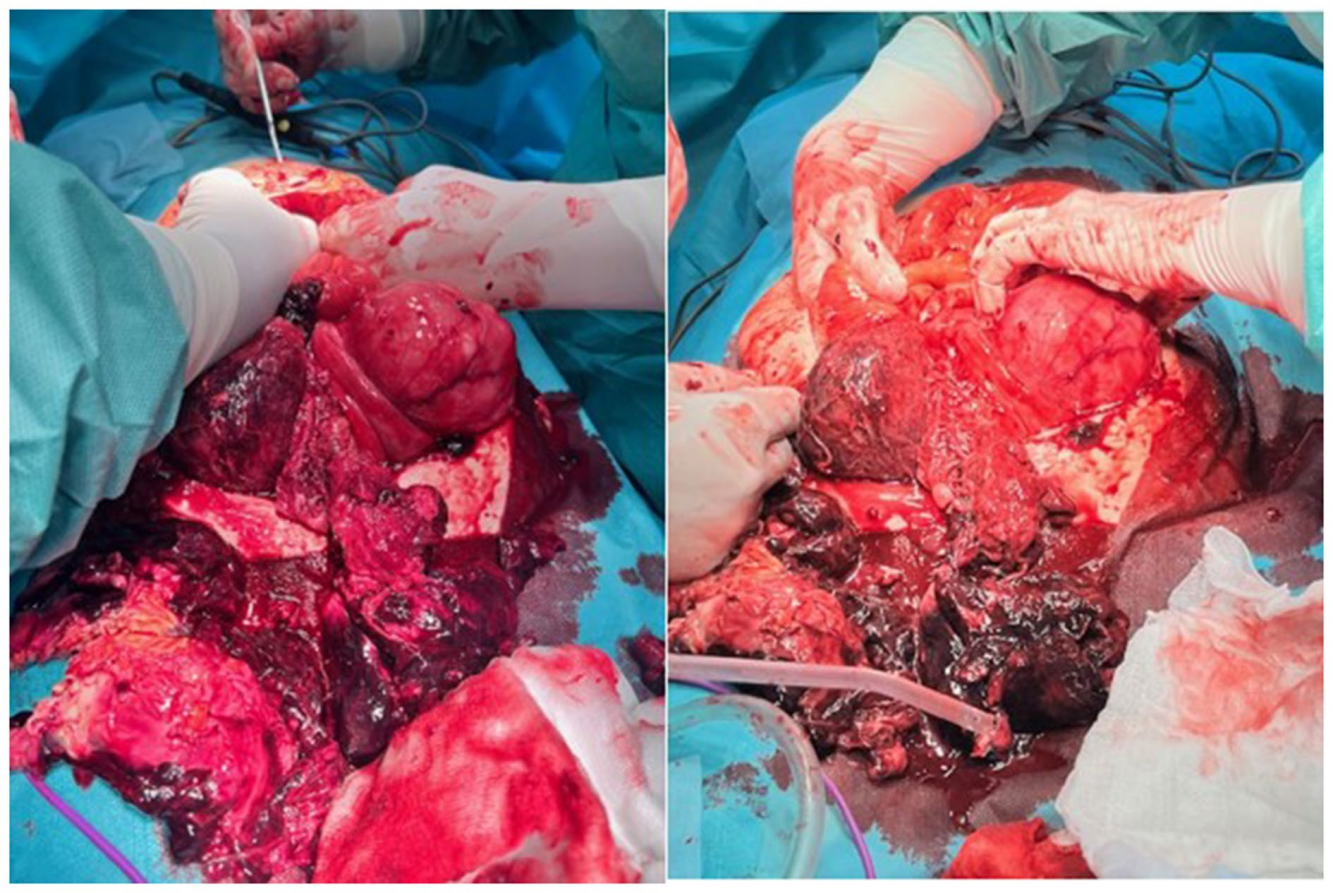
Infraumbilical laparotomy and exploration of the abdominal cavity, discontinuity of the anterior uterine wall with a large hematoma on the rupture spot.

Intraoperative findings revealed an enlarged uterus with a tumor mass, anterior wall rupture with focal necrosis, and hemorrhage with multiple urinary bladder peritoneal adhesions. Both the adnexa were morphologically unremarkable. A total abdominal hysterectomy with bilateral adnexectomy and omentectomy was performed (Figure 2).

Histopathological macroscopic findings showed both adnexa with a deformed uterus, 200 × 110 × 120 mm in diameter, weighing 1,200 g. On the right lateral anterior uterine wall, a 30 mm long defect of uterine tissue with rough edges was yellowish-red in color, distinctly softened, and 102 mm in diameter. On the uterine surface, there was a subserosal nodule, 83 mm in diameter, soft in consistency, with a ring-shaped field on the cross-section. The cavum was dislocated, and the endometrium was up to 3 mm thick in the uterine body section. The adnexa and cervix appeared normal. Processing methods: hematoxylin and eosin staining and immunohistochemistry (IHC).

**Figure 3:**
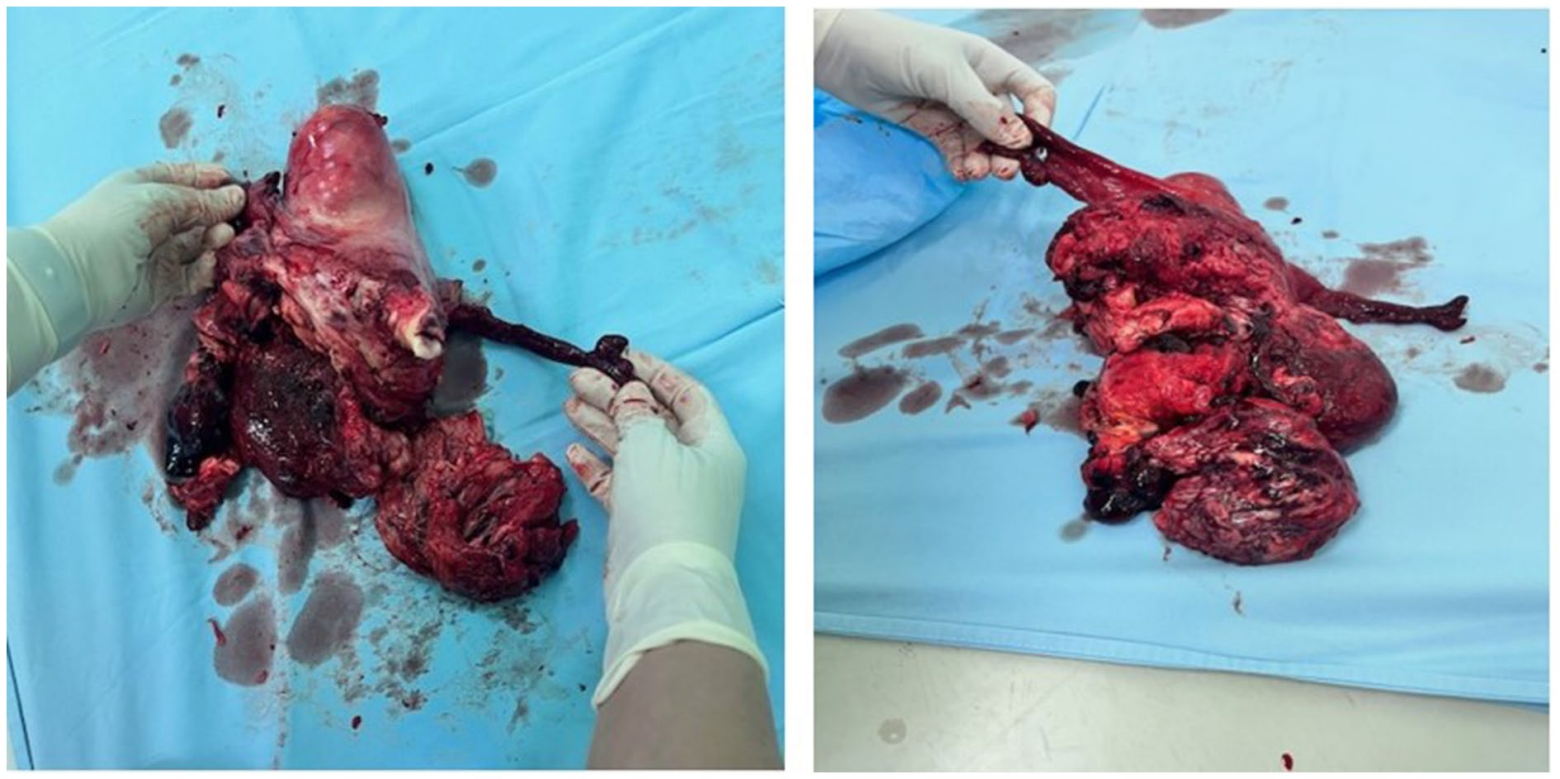
Uterus with both salpinges and tumor on the anterior wall, with a hematoma on the rupture spot with parts of the tumor.

The front uterine wall was infiltrated by a necrotic tumor mass with extensive fields of hemorrhage, mostly a swirling appearance, a very thin stroma, and markedly proliferated blood vessels. The tumor showed a fascicular-solid morphology, composed of strikingly polymorphic cells, mostly spindle-shaped and eosinophilic cytoplasm, with hypertrophic-hyperchromatic, partly bizarre nuclei, including visible multinucleated giant cell elements, with clearly visible increased mitotic activity. Tumor tissue with the same characteristics was seen in separately delivered tissue fragments and in tissue fragments labeled as “small intestine changes biopsy.” The blood vessel invasion was multifocal. Remnants of a benign mesenchymal smooth cell tumor were observed in the surrounding tissue. Immunohistochemically, tumor cells were diffusely positive for smooth muscle actin, desmin, caldesmon, calponin, p16, and cytokeratin AE1/AE3; approximately 30% of cells expressed p53 (measured twice), approximately 10% of cells expressed estrogen receptors (10%++, score 4), and were negative for CD10 and progesterone receptors. The proliferation index was remarkably high, and Ki-67 was expressed in approximately 75% of the cells (Figure 3).

Pathologist’s conclusion: The pathohistological picture of the tumor, along with the obtained immunophenotype, corresponded to malignant mesenchymal proliferation, which is a classical variant of uterine LMS with a high degree of malignancy.

**Figure 4:**
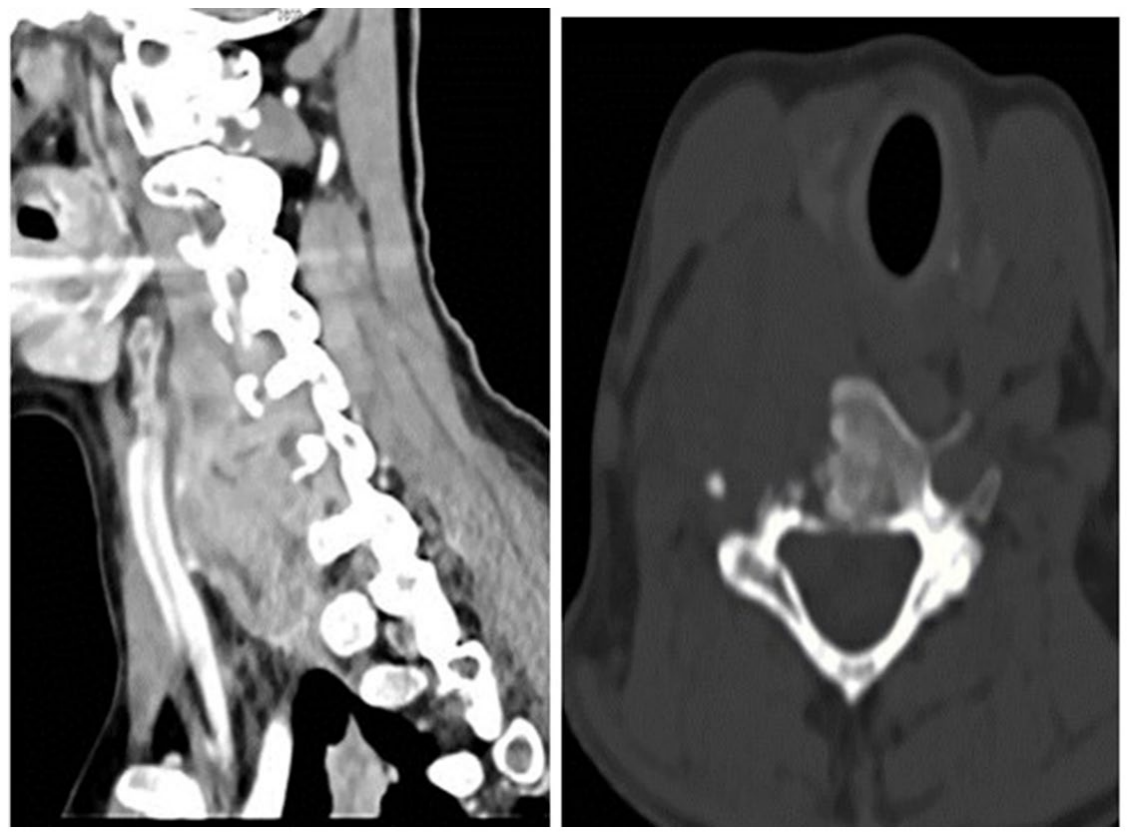
Right-sided neck mass.

Radiologists performed multiple diagnostic imaging studies that revealed lung, liver, colon, and peritoneal metastases. There was a right-sided neck colliquative mass (most likely metastasis), 75 × 40 × 35 mm in diameter, with C5 and C6 vertebral destruction and propagation to the spinal canal (Figure 4).

The patient was then referred to a multidisciplinary oncology advisory board. The patient had distant metastases; hence, the disease was classified as an IVB stage as per the International Federation of Gynecology and Obstetrics classification. The Oncology Advisory Board proposed chemotherapy, including adriamycin monotherapy.

## 3 Discussion

The strength of our study is the report of the unwanted outcome of the STUMP misdiagnosis and surgery outcome. This study has potential limitations: the effect estimates in the model are based on a retrospective observational study. There is also a lack of available data (from private clinic surgery and pathophysiological examination). Considering that the study is a case report, there is a lack of data that can lead to a conclusion that could be generalized. Therefore, future studies on this delicate topic are necessary.

This paper presents a 49-year-old woman with STUMP with non-specific symptoms, such as abdominal pain, pelvic pressure, abnormal uterine bleeding, and anemia (17). She had non-specific symptoms and belonged to an age group in which STUMP and LMS mostly occur (18). Our Clinical Center’s diagnostic imaging protocol for uterine tumors includes ultrasonography, MDCT, and MRI.

Ultrasound examination is the first step in the evaluation of uterine tumors; however, a limited field of view is a problem with large tumors. Some sonographic features, such as gross tumor size (>5 cm), highly vascularized mass, irregular outline, and necrotic areas, are more often linked to LMS and STUMP (11). Morphological uterine sonographic assessment (MUSA) has been proposed by consensus to describe the ultrasonographic features of the myometrium and uterine mass. According to the MUSA, STUMP shows higher color Doppler enhancement than that in benign masses due to higher intralesional and perilesional vascularization (11).

MDCT of the abdomen and pelvis is the first choice for urgent diagnostic imaging. It provides information on tumor size, extension, margins, structure (necrosis and hemorrhage), potential ascites, lymphadenopathy, and metastasis.

The absence of ionizing radiation, visualization of the uterine zonal anatomy, and multiparametric protocols make MRI the most useful imaging modality for evaluating uterine tumors (19).

There should be no difficulty for experienced radiologists to differentiate between typical leiomyoma (sharp, smooth margins, diffuse transverse relaxation time-weighted image hypointensity, no diffusion restriction–low diffusion-weighted imaging/apparent diffusion coefficient of water “blackout phenomenon,” and mild postcontrast enhancement) and LMS (irregular ill-defined margins, areas of necrosis and hemorrhage, diffusion restriction, and heterogeneous postcontrast enhancement) (19–21).

However, there is a large overlap between variant leiomyomas (atypical and degenerative), STUMP, and LMSs.

There are a few examples of malignancy scoring systems and MRI models for uterine tumor evaluation that assess tumor margins, transverse relaxation time, diffusion-weighted imaging/apparent diffusion coefficient of water (apparent diffusion coefficient of water value below a threshold), and contrast enhancement (22–25).

Thus far, there has been an average predictive value in the difference between these two groups, and a larger series of cases is necessary to confirm the reliability of the MRI models and scoring systems (21).

After the first laparoscopic surgery, the diagnosis of STUMP was made on the specimen after morcellation. Morcellated tumors can often be overdiagnosed or underdiagnosed by the pathologist because of the limitations of histological evaluation (26). Because of those limitations and the lack of exact pathological criteria, the combination of IHC and clinicopathological findings can be more helpful in determining the final diagnosis and risk of recurrence in STUMP (18).

In a previous study, IHC played a significant role in the differentiation between smooth muscle tumors and endometrial stromal tumors based on positive h-Caldesmon expression and CD10 negative expression; p16 and p53 were diffusely positive, indicating a poor prognosis (7).

Late molecular studies have focused on finding solutions for the preoperative diagnosis of LMS and STUMP and their differential diagnosis of benign uterine fibroids, seeking possible biomarkers based on molecular variations (3, 9). Analyses of gene mutations and chromosomal abnormalities revealed 21 upregulated genes and 74 downregulated genes with possible roles in LMS development (3, 27). These findings are not yet clinically applicable. However, in the era of omics, they are promising starting points, together with technical improvements in imaging diagnostics. Investigating the involvement of the PD1/PD-L1 axis checkpoint in the pathogenesis of mesenchymal tumors is another promising research field. Some studies have shown tumor PD-L1 expression in 70% of LMSs and 14% of atypical leiomyomas; however, no cases of STUMP or benign leiomyomas have shown PD-L1 expression (28).

All of these efforts to achieve a preoperative diagnosis of STUMP and LMS are very important in individualizing therapeutic approaches for each patient. The treatment of choice for STUMP is surgery, myomectomy, or hysterectomy with or without bilateral salpingo-oophorectomy (3, 6, 29). The surgical approach should be tailored based on the patient’s age, fertility-sparing wishes, and the size and location of the tumor. The laparoscopic approach is an acceptable choice if performed with “in bag” morcellation because of the peritoneum dissemination of malignant cells (3, 11, 30). It results in shorter hospitalization, less postoperative pain, less morbidity, and less adhesion formation (3, 30, 31). Total hysterectomy is the golden standard treatment, and preservation of ovaries could be considered in perimenopausal women, especially in stages I and II of the disease (2, 32).

## 4 Conclusion

STUMP are extremely rare and are mostly diagnosed in perimenopausal women, at an average age of 44 years (29, 33). Hematoperitoneum due to spontaneous rupture of a uterine tumor is even rarer (16). Our patient had no desire for fertility preservation but refused open surgery during her first hospitalization. There was no convincing preoperative evidence obtained by imaging studies, laboratory investigations, or clinical examination of the malignant potential of her uterine tumor. Therefore, the patient was unable to obtain adequate information about the potential risk of not accepting the proposed surgical treatment. Even after surgery, when a histopathological diagnosis of STUMP is obtained, a detailed explanation of the unpredictable course of the disease should be provided to the patient. For patients who have achieved fertility or have no desire to become pregnant, total hysterectomy with or without bilateral salpingo-oophorectomy should be the treatment of choice to avoid possible recurrence (4, 34–36). In our case, recurrence and malignant progression occurred within a short period. This could be linked to cellular diffusion in the peritoneal cavity owing to unprotected electrical morcellation (4). Although there is no established follow-up protocol for patients with STUMP, follow-up may include a physical examination and blood tests every 6–12 months with additional imaging tests on an as-needed basis (37).

## 5 Patient perspective

My first doctor told me that I most likely had leiomyoma, and when I came to the Clinical Center for the first time, there was no convincing evidence of the malignant nature of the tumor; therefore, I decided to undergo minimally invasive surgery instead of open hysterectomy. Even after the first surgery, there was no strong evidence to convince me to undergo another radical surgery. I was worried about the long hospital stay, pain after surgery, and scarring.

## Data availability statement

The raw data supporting the conclusions of this article will be made available by the authors, without undue reservation.

## Ethics statement

Written informed consent was obtained from the participant for the publication of any potentially identifiable images or data included in this article.

## Author contributions

MB-I: Writing – original draft, Writing – review & editing, Conceptualization, Validation, Visualization. II: Writing – original draft, Conceptualization, Data curation, Formal analysis, Funding acquisition, Investigation, Methodology, Project administration, Resources, Software, Supervision, Validation, Visualization. AD: Project administration, Resources, Supervision, Writing – review & editing. SM: Data curation, Methodology, Validation, Software, Writing – review & editing. NJ: Investigation, Methodology, Validation, Writing – review & editing. DR: Data curation, Software, Visualization, Writing – original draft. NA: Conceptualization, Data curation, Methodology, Writing – review & editing. TN-T: Conceptualization, Data curation, Formal analysis, Funding acquisition, Investigation, Methodology, Project administration, Resources, Software, Supervision, Validation, Visualization, Writing – review & editing. GB: Conceptualization, Methodology, Validation, Writing – review & editing. AP: Data curation, Investigation, Writing – original draft. AM: Investigation, Project administration, Validation, Writing – original draft. AN: Conceptualization, Data curation, Formal analysis, Funding acquisition, Investigation, Methodology, Project administration, Resources, Software, Supervision, Validation, Visualization, Writing – review & editing.
